# Olive leaf‐derived PPAR agonist complex induces collagen IV synthesis in human skin models

**DOI:** 10.1111/ics.12742

**Published:** 2021-11-04

**Authors:** George P. Majewski, Smrita Singh, Krzysztof Bojanowski

**Affiliations:** ^1^ Contrast Product Development Walnut California USA; ^2^ Creative Bioinformatics and Science Morna District Bijnor, Uttar Pradesh India; ^3^ Sunny BioDiscovery Santa Paula California USA

**Keywords:** cell culture, collagen IV, computer modelling, dermal–epidermal junction, olive leaf extract, skin barrier

## Abstract

**Introduction:**

Peroxisome proliferator‐activated receptor (PPAR) agonists are known to modulate the synthesis of dermal lipids and proteins including collagens. Olive (*Olea europaea*) leaves have been reported to contain PPAR‐binding ligands. Collagen IV, a major dermal‐epidermal junction (DEJ) protein, degrades with both age and disease. Here, we report the formulation of a novel multi‐ligand complex, Linefade, and its effects on collagen IV synthesis.

**Methods:**

Linefade prepared from the leaves of *Olea europaea* contains 2% w/w plant extract solids dissolved in a mixture of glyceryl monoricinoleate and dimethyl isosorbide. In silico docking was performed with PPAR‐α (PDB ID: 2P54). Linefade was evaluated for PPAR‐α‐dependent transcription in a luciferase reporter assay system. Cell viability and collagen IV levels in human dermal fibroblast cultures were measured using the MTT method and ELISA assay, respectively. Transcriptome analysis was conducted on a full‐thickness reconstituted human skin (EpiDermFT) model. Ex vivo cell viability and collagen IV immunostaining were performed on human skin explants.

**Results:**

In silico docking model of the major constituents (oleanolic acid and glyceryl monoricinoleate) produced a co‐binding affinity of −6.7 Kcal/mole. Linefade significantly increased PPAR‐α transcriptional activity in CHO cells and collagen IV synthesis in adult human dermal fibroblasts. Transcriptome analysis revealed that 1% Linefade modulated the expression of 280 genes with some related to epidermal differentiation, DEJ, PPAR, Nrf2 and retinoid pathways. An ex vivo human explant study showed that 1% Linefade, delivered via a triglycerides excipient, increased collagen IV levels along the dermal–epidermal junction by 52%.

**Conclusion:**

In silico modelling and in vitro and ex vivo analyses confirmed Linefade‐mediated activation of PPAR‐α and stimulation of collagen IV synthesis.

## Graphical abstract

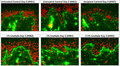

## Résumé

**Introduction:**

Les agonistes du récepteur activé par les proliférateurs de peroxysomes (PPAR) sont connus pour moduler la synthèse des lipides cutanés et des protéines du derme, y compris des collagènes. Il a été signalé que les feuilles d'olivier (Olea europaea) contiennent des ligands de liaison aux PPAR. Le collagène IV, une protéine majeure de la jonction dermo‐épidermique (DEJ), se dégrade avec l'âge et la maladie. Nous rapportons ici la formulation d'un nouveau complexe multi ligand, Linefade, et ses effets sur la synthèse du collagène IV.

**Méthodes:**

Le complexe Linefade préparé à partir des feuilles d'Olea europaea contient 2 % p/p de solides d'extraits végétaux dissous dans un mélange de monoricinoléate de glycéryle et d'isosorbide de diméthyle. Un docking in silico a été réalisé avec PPAR‐α (PDB ID : 2P54). Linefade a été évalué pour la transcription dépendante du PPAR‐α dans un système de test rapporteur à la luciférase. La viabilité cellulaire et les niveaux de collagène IV dans les cultures de fibroblastes dermiques humains ont été respectivement mesurés en utilisant la méthode MTT et le test ELISA. L'analyse du transcriptome a été réalisée sur un modèle de peau humaine reconstitué sur toute son épaisseur (EpiDermFT). La viabilité cellulaire ex vivo et l'immunomarquage du collagène IV ont été réalisés sur des explants de peau humaine.

**Résultats:**

Le modèle de docking in silico des principaux constituants (acide oléanolique et monoricinoléate de glycéryle) a produit une affinité de liaison conjointe de ‐6,7 Kcal/mole. Linefade a augmenté de manière significative l'activité transcriptionnelle du PPAR‐α dans les cellules CHO et la synthèse du collagène IV dans les fibroblastes dermiques humains chez les personnes adultes. L'analyse du transcriptome a révélé que 1% de Linefade modulait l'expression de 280 gènes dont certains étaient liés à la différenciation épidermique, à la DEJ, au PPAR, à la voie Nrf2 et aux voies rétinoïdes. Une étude ex vivo sur des explants humains a montré que 1% de Linefade, délivré via un excipient de triglycérides, augmentait de 52% les niveaux de collagène IV le long de la jonction dermo‐épidermique.

**Conclusion:**

La modélisation in silico et les analyses in vitro et ex vivo ont confirmé l'activation du PPAR‐‐ α et la stimulation de la synthèse du collagène IV par Linefade.

## INTRODUCTION

Collagen IV is a major anchoring protein found mostly in lamina densa of the dermal–epidermal junction of human skin. As a major constituent of the basement membrane, collagen IV provides a three‐dimensional scaffold with other macromolecules such as laminins, fibronectin and heparan sulphate proteoglycans in promoting anastomosis between the epidermis and the underlaying dermis. It also plays a significant role in wound healing and embryogenesis and promotes cell adhesion and proliferation [1, 2, 3]. Six human genes code for collagen IV chains, that is COL4A1, COL4A2, COL4A3, COL4A4, COL4A5 and COL4A6 [4]. Type IV collagen anomalies are associated with several diseases, including Alport and Goodpasture's syndromes, several rheumatological and dermatological diseases. Major changes and degradation of collagen IV are linked to subepidermal blistering diseases, including bullous pemphigoid. Autoimmune subepidermal bullous diseases (AISBDs) lead to irregular collagen type IV immunohistochemical staining, which is a useful technique for diagnosis of AISBDs [5] and classification of cutaneous autoimmune subepidermal blistering disorders [6]. Blistering diseases are rare skin diseases that occur when the immune system attacks the skin and mucous membranes.

Skin ageing occurs due to both intrinsic and extrinsic factors. Intrinsic ageing is a physiological process that leads to thin, dry skin, fine wrinkles and gradual dermal atrophy. Air pollution, smoking, poor nutrition and sun exposure are extrinsic ageing factors that result in crepey skin, loss of elasticity, increase in sagging and rough‐textured appearance [7, 8]. With advancing age, there is a decrease in the collagen IV expression of matured dermal fibroblasts [9]. Furthermore, a previous immunohistochemical study revealed that significant reduction in the levels of collagen IV in non‐photodamaged skin of older individuals (mean age: 71.5 years) compared with the younger individuals (mean age: 23.4 years) [10].

Peroxisome proliferator‐activated receptors (PPARs) are nuclear hormone receptors similar to retinoid receptors. They act as transcription factors that modulate the expression of target genes by binding to specific DNA sequences. This ligand‐activated transcription has been shown to regulate activities across various skin cell types and metabolic activities. During its activation, PPAR‐alpha heterodimerizes with retinoid X receptor beta (RXRβ), and together, these receptors activate the acyl‐CoA oxidase gene promoter [11]. Acyl‐CoA oxidase metabolizes very long‐chain fatty acids via oxidative processes.

The aldo–keto reductase (AKR) superfamily metabolizes carbohydrates, steroids, prostaglandins, endogenous aldehydes, ketones and xenobiotic compounds. AKR1B10 mediates retinoic acid homeostasis by diminishing the cellular levels of retinoic acids and reducing the availability of retinaldehydes through the conversion of retinaldehydes into retinols [12, 13].

Olive leaf and olive leaf extract are known for beneficial health properties such as antioxidant, anti‐inflammatory and anti‐bacterial effects. The leaves contain a complex variety of bioactive substances, including phenolic compounds, triterpenic acids, iridoids and sugars, such as oleanolic and maslinic acids, oleuropein, oleuropeoside, ligustroside, hydroxytyrosol and mannitol [14]. Both oleanolic acid and oleuropein have been reported to activate the PPAR‐alpha receptor [15, 16]. Furthermore, oleanolic acid also demonstrates dual agonist action on two isoforms of PPAR γ/α and a potent inhibitory action against AKR1B10 [17, 18, 19].

In other PPAR‐related skin care applications, 10‐hydroxystearic acid, a vegetable oil‐derived synthetic fatty acid, has been reported to stimulate collagens I and III in cell culture models and improves facial age spots and conspicuous pores in in vivo [20]. However, there have been limited number of studies, which explore PPAR activation induced by complex mixtures containing more than one ligand [21, 22, 23].

Different therapeutic modalities have been proposed for blistering diseases; however, all of them have their own limitations and side effects on long‐term use. A previous study observed that agonists of PPAR can act as antagonists for AKR1B10 [24]. Therefore, the identification of a mixture of compounds targeting PPAR activation with minimal or no side effects for management of ageing skin and blistering diseases could be of value for formulating both topical skin care and therapeutic products.

In skin care applications, the availability of ingredients that are known to induce synthesis collagen IV is limited. A compound extracted from Psoralea corylifolia called bakuchiol has been shown to upregulate types I and IV collagen in a DNA microarray study and also showed stimulation of type III collagen in a mature fibroblast model [25].

Keeping this in view, this study was conducted to examine whether a complex mixture of PPAR ligands (Linefade) may cooperatively bind in the PPAR‐α cavity to activate gene expression and lead to collagen IV biosynthesis. To explore this possibility, an in silico technique was employed to model two of the main constituents (oleanolic acid and glyceryl monoricinoleate) in a co‐binding arrangement. In vitro cell culture and transcriptome analysis were used to confirm PPAR activation and gene expression. Stimulation of collagen IV synthesis in cultured human dermal fibroblasts was assessed using an ELISA assay. Further corroboration of collagen IV synthesis was examined ex vivo in human skin explants using immunostaining and image analysis.

## MATERIALS AND METHODS

### Materials

Dimethyl sulfoxide (DMSO), caprylic/capric triglyceride (CCT), magnesium ascorbyl phosphate (MAP) and GW590735 were obtained from various suppliers. Indigo Biosciences Reporter Assay System was used for luciferase reporter assay.

### Preparation of linefade

Linefade was extracted from the powdered leaves of Olea europaea via an alcoholic extraction. The wet cake was dried into a powder, and oleanolic acid was assayed by HPLC method to not less than 80.0% w/w on dried basis. The remaining 20% contained a variety of alcohol‐soluble compounds commonly found in olive leaves. Next, 2% w/w of the dried solid powder was resolubilized with a 1:1 solvent mixture of glyceryl monoricinoleate and dimethyl isosorbide. Heat and agitation were applied to achieve a clear solution without any precipitate. The resulting composition contains not less than 1.6% oleanolic acid and 0.4% of undetermined compounds derived from olive leaves.

### In silico modelling technique and validation

The 3D structure of PPAR‐α (PDB ID: 2P54), bound with SRC1 peptide and GW590735, was considered as the known PPAR‐α activator to determine the active sites. The retrieved structure of PPAR‐α from the RSCB database (PDB: 2P54) was optimized by the addition of hydrogens, removal of all water molecules and optimizing hydrogen bonds using Discovery Studio Visualizer (ver 2.5). The optimized structure was defined as target macromolecule in Autodock Vina Wizard of PyRx (ver 0.8). GW590735 and major constituents of Linefade were docked to the ligand‐binding domain. The docking experiment was run in the PyRx software workspace on default parameters of the number of generations and energy evaluation for ten steps of the run. The predicted binding affinity was expressed as kcal/mol. While docking, different binding poses were generated among which the best and top‐ranked binding pose was selected for visual inspection using Discovery Studio Visualizer (ver 2.5).

### In vitro cell studies

#### PPAR transcription

Linefade was stored at room temperature and was diluted in DMSO or distilled sterile water at 10 mg/ml. The stock solution was further diluted with compound screening medium as per kit manufacturer's instructions (Indigo Biosciences, State College, PA, cat. # IB00111‐32, Technical Manual version 7.2). Three concentrations (4, 20 and 100 µg/ml) were tested in duplicates on CHO cells transfected with the PPAR‐α receptor‐controlled bioluminescent protein for ~20 h. GW590735 (0.143 µg/ml) was the positive control. The chemoluminescent signal, proportional to the PPAR‐α‐driven promoter activation, was quantified using Thermo Scientific Luminoskan Ascent Microplate Luminometer. This instrument has passed DLReady™ validation at Promega Corporation.

#### Cell viability and type IV collagen output in the adult human dermal fibroblasts

##### Cell line and cell culture

Human dermal fibroblasts (HDFs) from adult skin (early passage, cat.# 106‐05aCell Applications, San Diego, CA) were maintained in DMEM‐based medium containing 10% FBS medium and 1% penicillin‐streptomycin at 37°C in a humidified, 95% air/5% CO2. All colorimetric measurements were performed using Molecular Devices (San Jose, CA) microplate reader MAX190 and SoftMax3.1.2PRO software.

##### Measurement of cell viability

The effect of Linefade on cell viability was determined using the MTT assay, which measures the activity of mitochondrial dehydrogenases, such as succinate dehydrogenase, implicated in the respiratory electron transport chain in mitochondria [26]. Linefade was dissolved at 20 mg/ml in DMSO and further diluted using distilled water. Samples were added in triplicates to confluent adult human dermal fibroblasts. The positive control contained magnesium ascorbyl phosphate (MAP). After 72 h, the experiment was terminated, and the effect of Linefade on mitochondrial metabolism was measured.

##### Quantitative detection of collagen IV

Type IV collagen was quantified in the cell culture medium (soluble fraction) via sandwich ELISA assay after treatment with Linefade for 72 h, using unlabelled anti‐type IV antibody (cat.#1340‐01) for capture, followed by biotinylated anti‐type IV collagen antibody (cat.# 1340‐08), streptavidin‐HRP and TMB reagents from SouthernBiotech (Birmingham, AL).

### Assay on reconstituted skin substitutes

EpiDermFT tissues (MatTek; Ashland, MA) were equilibrated overnight; then, samples were added in duplicates at 3 mg/cm^2^ with a positive displacement pipette and were spread evenly on top of the tissues. Sterile distilled water was the negative control. After 24 h incubation, RNA was extracted and purified with RNeasy Mini Kit cat.# 74104 from Qiagen (Germantown, MD), using QiaCube Connect robotic station (Qiagen). Purified total RNA was assessed at 260 and 280 nm with NanoDrop Lite (Thermo Fisher Scientific, Waltham, MA).

Isolated RNA samples were sent on dry ice to Thermo Fisher Scientific Microarray Research Services Laboratory (Santa Clara, CA) for transcriptome profiling using Clariom S cartridge on GeneTitan™ Microarray System instrumentation. The resulting CHP files containing probe set analysis results generated with Affymetrix software were uploaded, and differential gene expression as well as functional interaction networks were analysed using the TAC software version 4.0.2.15 (Applied Biosystems by Thermo Fisher).

### Ex vivo study

The ex vivo study was conducted as per the Good Laboratory Practices (Decree of August 10, 2004), as well as in compliance with the validated procedures and SOP of Laboratoire BIO‐EC. Forty‐two explants of an average diameter of 11 mm (±1 mm), including 15 delipidated explants, were prepared from an abdominoplasty coming from a 28‐year‐old woman (reference P2302‐AN28) with a skin phototype V. These skin tissues were obtained from surgical residues and are in compliance with the Declaration of Helsinki and the article L.1243‐4 of the French Public Health Code. The delipidated set was utilized for examining ceramide synthesis (to be reported elsewhere). Explants were placed in BEM culture medium (BIO‐EC’s Explants Medium) at 37°C in a humid, 5% CO_2_ atmosphere. The Linefade complex was solubilized in caprylic/capric triglyceride (CCT) at 1% and 2.5% v/v concentrations. The study samples were divided into four groups: untreated control, excipient control, test 1 and test 2. The untreated control group was a blank. The excipient control group was treated with CCT alone. Test 1 and test 2 groups were treated with 1% and 2.5% Linefade, respectively. CCT with or without Linefade was topically applied to skin explants at days 0 and 2 on the basis of 2 μl per explant (2 mg/cm^2^) and spread using a small spatula. On day 2, half of the culture medium was replaced by fresh medium. On day 0, the untreated control explants were collected and cut into two parts: One part was fixed in buffered formalin solution for 24 h, and the other part was frozen at −80°C. On day 3, three explants from each batch were collected and processed in the same way as the untreated control.

#### Tissue morphology

The explants were fixed for 24 h in buffered formalin, dehydrated and then impregnated in paraffin using a Leica PEARL dehydration automat. The samples were embedded using a Leica EG 1160 embedding station. Next, 5‐μm‐thick sections were made using a Leica RM 2125 Minot‐type microtome, and the sections were mounted on Superfrost^®^ histological glass slides. The tissue morphology of the epidermal and dermal structures was assessed through microscopic observation of formalin‐fixed paraffin‐embedded (FFPE) skin sections after Masson's trichrome staining, Goldner variant. The degree of staining was assessed by microscopic observation.

#### Quantification of collagen type IV

The frozen explants were cut into 7‐μm‐thick sections using a Leica CM 3050 cryostat. The sections were then mounted on Superfrost^®^ plus silanized glass slides. The microscopic observations were realized using a Leica DMLB, an Olympus BX43, or BX63 microscope. Pictures were digitized with a numeric DP72 or DP74 Olympus camera with cellSens storing software. Collagen IV immunostaining was performed on frozen sections with a monoclonal anti‐collagen IV antibody (Dako, ref. M 0785, clone CI22) diluted at 1:50 in PBS‐BSA 0.3%‐Tween 20 and incubated for 1 h at room temperature and revealed by AlexaFluor488 (Life technologies, ref. A11008). The nuclei were counterstained with propidium iodide. The immunostaining was assessed by microscopic examination and image analysis. All the images were analysed using Cell^D software.

### Statistical analysis

All data are expressed as Mean ± Standard Deviation (SD) or Mean ± Standard Error of Mean (SEM). The differences between control and treatment groups were evaluated by Student's double‐tailed *t*‐test. Statistically, significant variation was defined as ≥20% variation from the control with a *p*‐value < 0.05.

## RESULTS

### In silico modelling

**FIGURE 1:**
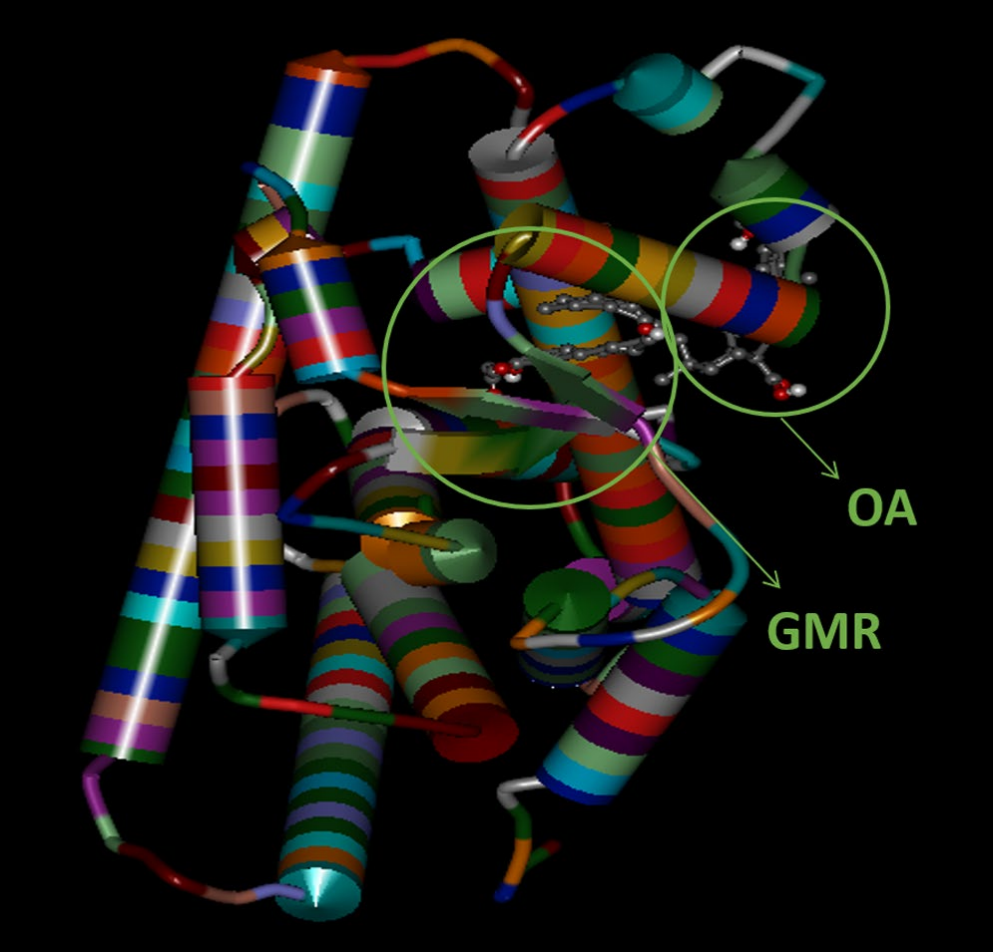
Major constituents of Linefade—oleanolic acid (OA) and glyceryl monoricinolate (GMR)—docked in the active site of PPAR‐α (PDB ID: 2P54), highlighting cooperative interactions

PPAR‐α (PDB ID: 2P54) contains 16 helices, 4 beta‐strands, 11 bends and 16 turns (Sierra et al., 2007). GW590735 occupies the binding pocket formed by the helices, bends and beta‐strands. The binding site of GW590735 in the ligand‐binding pocket of PPAR‐α is composed of the amino acid residues CYS‐275, CYS‐276, GLN‐277, THR‐279, ALA‐333, MET‐330, MET‐355, SER‐280, TYR‐314, HIS‐440 and TYR‐464. GW590735 formed hydrogen bonds with SER‐280, TYR‐314, HIS‐440 and TYR‐464 [27]. The binding affinity of GW590735 and major constituents of Linefade with target receptor (PDB ID: 2P54) was observed to be in the range of −5.7 Kcal/mole to −10.4 Kcal/mole (Figure 1 and Table 1).

**TABLE 1:** Binding affinity of major constituent of Linefade and GW590735 with PPAR‐α and their hydrogen bond interactions at the active site. Bold‐typed residues represent common interacting residues with GW590735

Compound name	Binding affinity (−Kcal/mol)	Amino acid interacting residues at a distance criterion of 5Ȧ	Number of common amino acid interacting residues with GW590735
GW590735 (synthetic PPAR‐α agonist standard)	−10.4	SER‐280, TYR‐314, TYR‐464, HIS‐440, CYS‐275, CYS‐276, GLN‐277, THR‐279, ALA‐333, MET‐330, MET‐355	_
Oleanolic acid	−5.7	HIS‐274, CYS‐278, TYR‐334, HIS‐47, LEU‐254	0
Glyceryl monoricinolate	−6.1	THR‐279, GLU‐21, CYS‐275, CYS‐276, SER‐280, ARG‐271, ILE‐272, PHE‐273, HIS‐274, THR‐246, LEU‐247, CYS‐248, MET‐249, ALA‐250, GLU‐251	4
Oleanolic acid + Glyceryl monoricinolate	−6.7	CYS‐276, SER‐280, LEU‐254, CYS‐275, THR‐279, ILE‐272, MET‐330, LEU‐331, MET‐355, GLN‐277, THR‐283, LEU‐247, ALA‐250, GLU‐251	7

### In vitro cell studies

#### PPAR transcription

**FIGURE 2:**
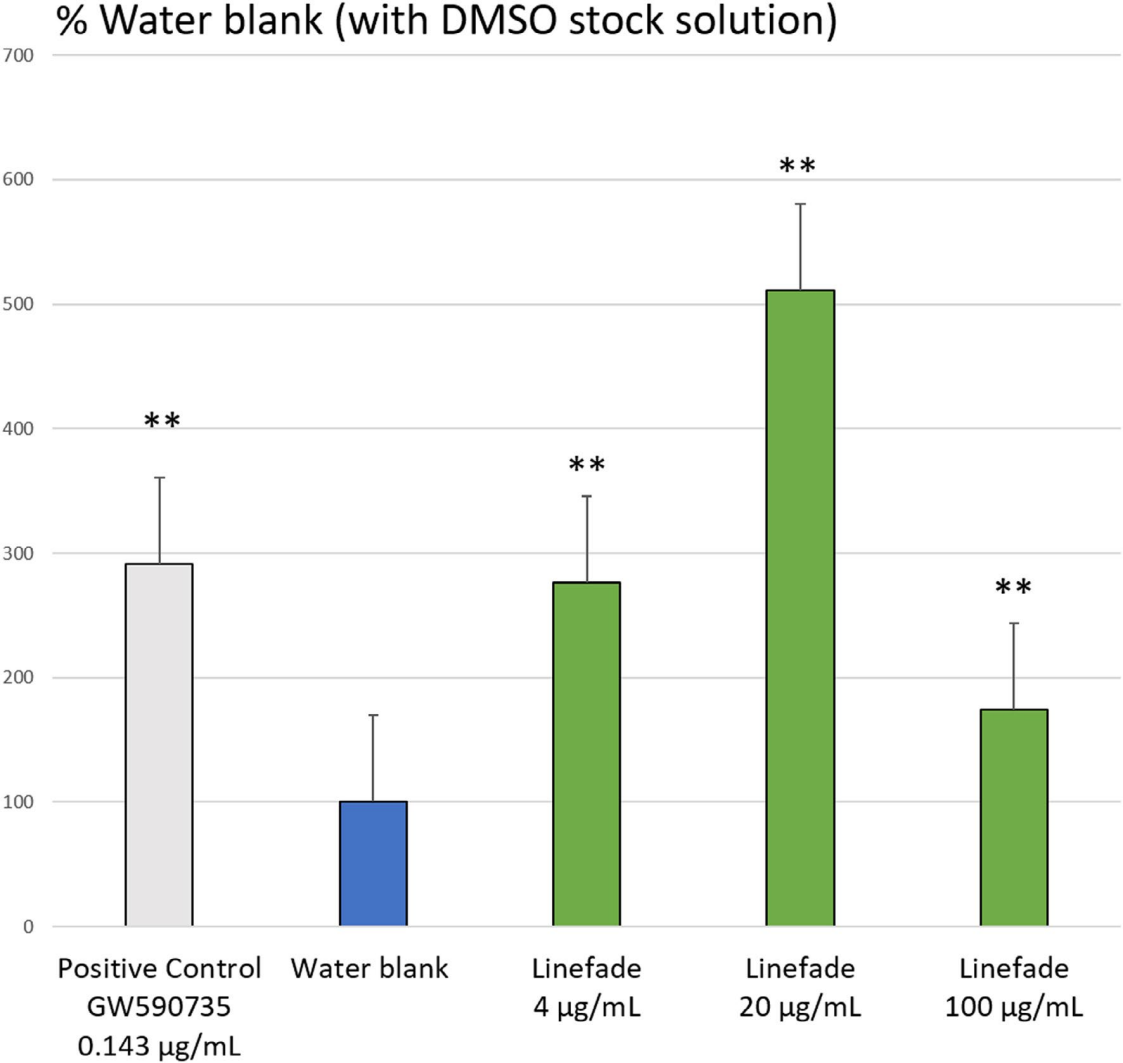
Degree of transcriptional activation of Linefade by PPAR‐α depicted by the Reporter Assay System with DMSO‐prepared stock solution of Linefade. Data are presented as Mean values ± SEM, *n* = 16; **p‐value < 0.01 vs. water blank [Colour figure can be viewed at wileyonlinelibrary.com]

**FIGURE 3:**
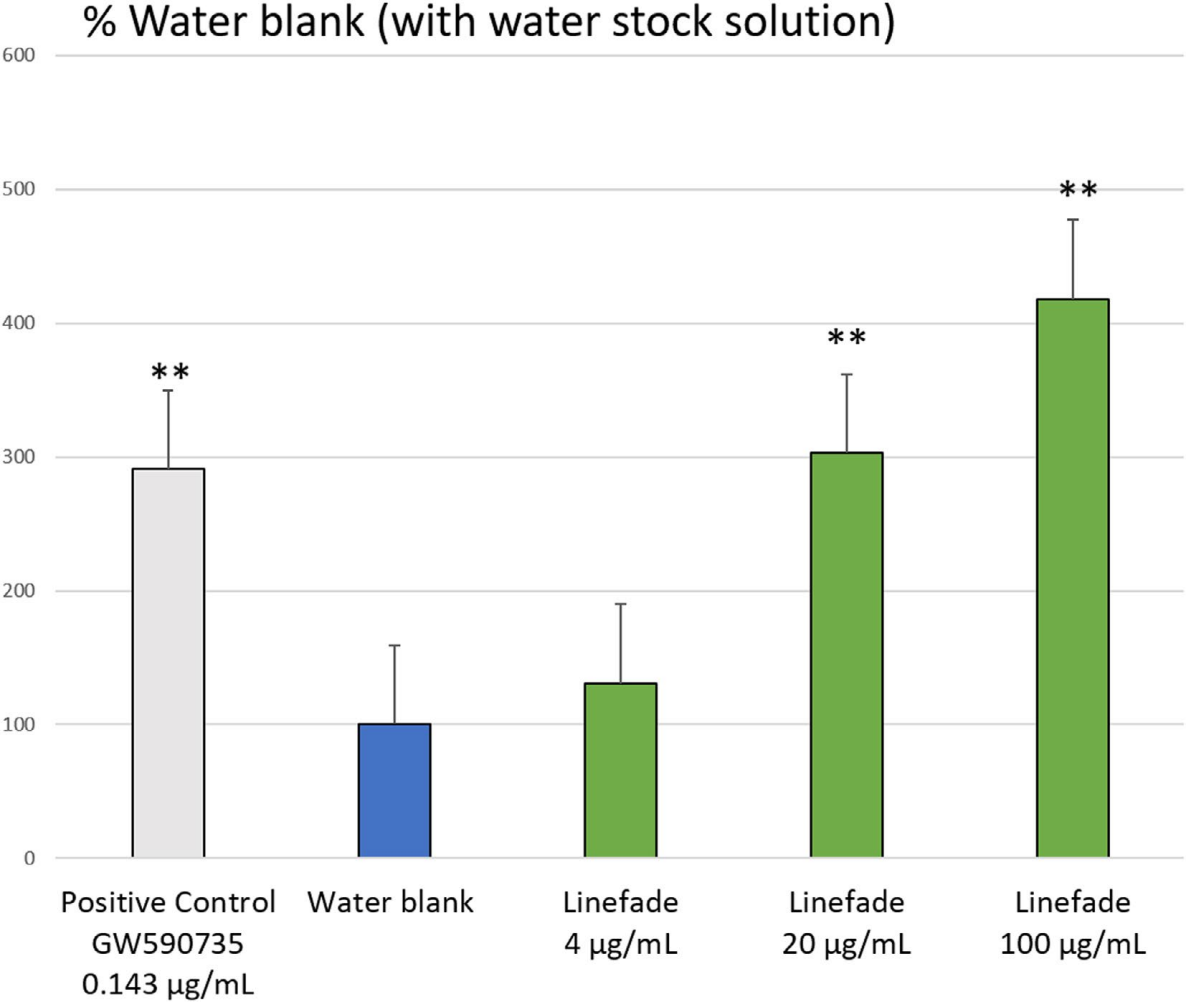
Degree of transcriptional activation of Linefade by PPAR‐α in the Reporter Assay System with water‐prepared stock solution of Linefade. Data are presented as Mean values ± SEM, *n* = 16; **p‐value < 0.01 vs. water blank [Colour figure can be viewed at wileyonlinelibrary.com]

Linefade stimulated the activation of transcription of the luciferase reporter gene under the PPAR‐α‐controlled promoter by 511% (*p*‐value = 0.000) at a 20 µg/ml dilution when applied from a DMSO stock solution. When applied from a water stock solution, Linefade stimulated the activation of transcription of the luciferase reporter gene under the PPAR‐α‐controlled promoter in a dose‐dependent manner with an increase of 418% occurring at a 100 µg/ml dilution. DMSO aids the dispersion of lipid‐soluble Linefade in this cell culture model but is not required to produce a significant activation. The positive control GW590735 (0.143 µg/ml) strongly upregulated the expression of the reporter gene (*p*‐value = 0.001) indicating experimental reliability (Figures 2 and 3).

#### Cell viability and collagen IV of human dermal fibroblasts (HDFs)

**FIGURE 4:**
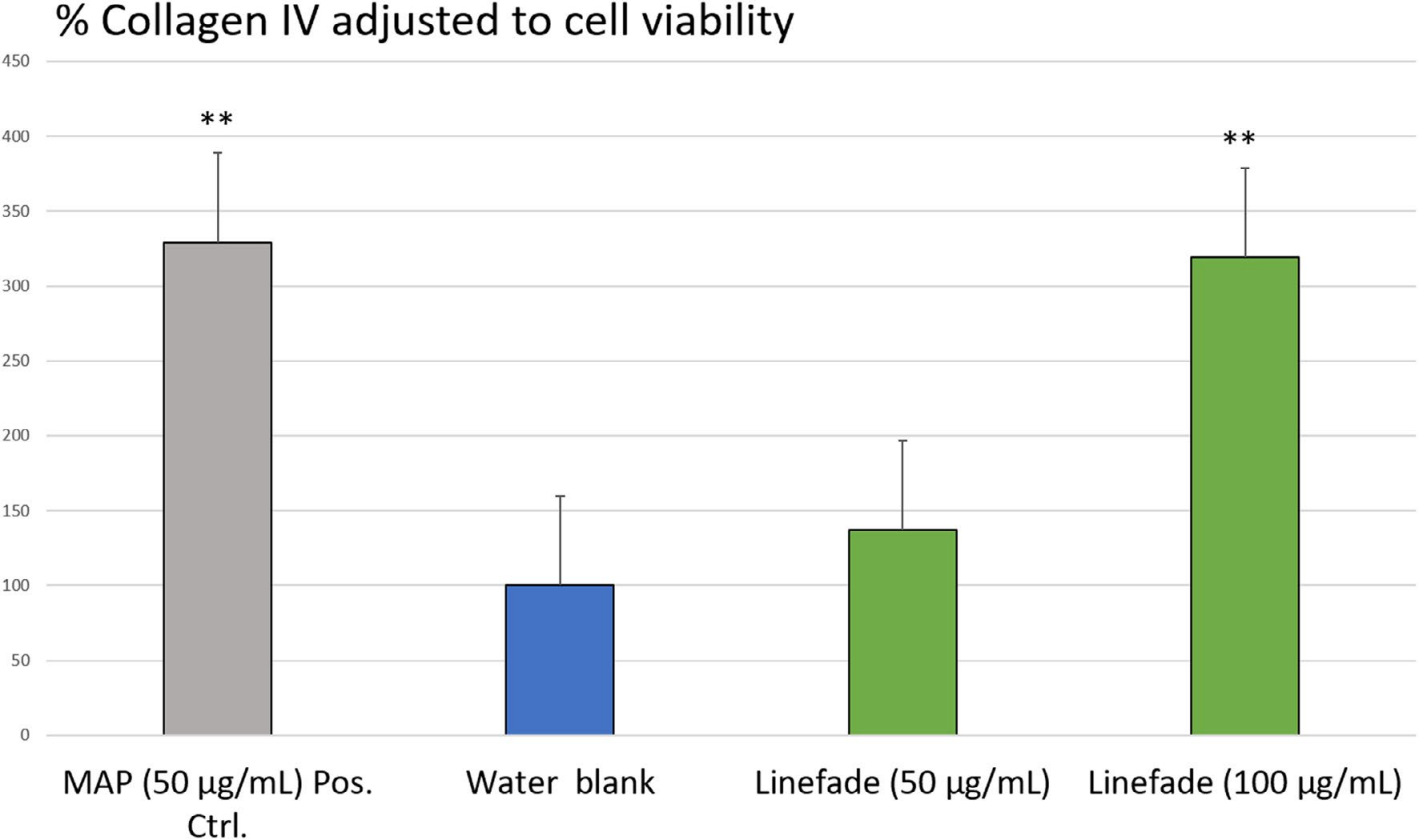
Dose‐dependent stimulatory effect of Linefade on the synthesis of collagen type IV expressed as percentage of water control, wherein 50 µg/ml MAP is a positive control. Data are presented as Mean values ± SEM, *n* = 6; **p‐value < 0.01 vs. water blank [Colour figure can be viewed at wileyonlinelibrary.com]

Cell viability was examined to exclude cytotoxic concentration of Linefade. Linefade did not show any cytotoxic effect on HDFs at concentrations of up to 200 μg/ml compared with water‐treated control. Linefade significantly stimulated type IV collagen output, being the most active at a concentration of 100 μg/ml. It was observed that treatment with 100 μg/ml Linefade led to a 319% increase in collagen IV level compared with the water‐treated control (*p*‐value = 0.000) (Figure 4).

### Transcriptome analysis

Differential expression comparing a water blank (five samples) and Linefade (two samples) was processed with TAC 4.0 software at a fold change of either >2 or <−2 and p‐value <0.05. Total number of genes in the data set was 21 448. Of these, 280 (1.31%) genes passed the filter criteria, among which 76.79% genes were upregulated and 23.21% genes were downregulated. Activity in relevant pathways was assessed with WikiPathways by significance and count in Table 2.

**TABLE 2:** Gene expression after treatment with water vs. Linefade assessed using Transcriptome Analysis Console 4.0.2.15 under filter criterion of fold change >2 or <−2 and *p*‐value <0.05

WikiPathways	Path significance	Up	Down
NRF2	1.81	CES4A, EGR1	HSPA1A, PGD, SLC39A2, SLC39A14
Nuclear receptor meta pathway	1.03	CES4A, EGR1	CDK4, SLC39A2, SLC39A14, ACOX1, PGD, HSPA1A
PPAR‐alpha pathway	0.51	‐	CKD4
PPAR signalling pathway	1.82	SORBS1	MMP1, ACOX1, MMP1
PPAR‐gateway pathway	1.64	‐	PLIN2, PCK2
PPAR‐gamma pathway	1.64	‐	PLIN2, PCK2
Retinoid metabolism and transport	0.38	‐	RDH11
Integrin‐mediated cell adhesion	1.27	SORBS1, CAPN3	ITGAE, CAPN1
Matrix metalloproteinases	1.64	‐	MMP1, MMP3

Genes related to epidermal differentiation (keratinocyte cornification), retinoid metabolism and collagen degrading MMPs were identified as shown in Table 3.

**TABLE 3:** Gene expression after treatment with water vs. Linefade assessed using Transcriptome Analysis Console 4.0.2.15 under filter criterion of fold change >1.5 or <−1.5 and *p*‐value <0.05

Fold Change	*p*‐value	Gene symbol	Description	Comments
Cornified envelope precursor genes				
6.99	0.0263	LCE1D	Late cornified envelope 1D	Late cornified envelope (LCE) genes within the epidermal differentiation complex
4.52	0.0166	LCE1F	Late cornified envelope 1F	
4.26	0.0249	LCE1A	Late cornified envelope 1A	
4.14	0.0286	LCE1B	Late cornified envelope 1B	
3.07	0.0471	LCE1C	Late cornified envelope 1C	
2.51	0.0481	LCE2D	Late cornified envelope 2D	
2.33	0.0181	LCE5A	Late cornified envelope 5A	
2.8	0.0358	S100A7A	S100 calcium binding protein A7A	S100A7 (Psoriasin) interacts with epidermal fatty acid‐binding protein and localizes in focal adhesion‐like structures in cultured keratinocytes [28]
1.61	0.0127	S100A7	S100 calcium binding protein A7	
2.26	0.0063	S100A12	S100 calcium binding protein A12	
2.51	0.0193	SPRR3	Small proline‐rich protein 3	The small proline‐rich proteins constitute a multigene family of differentially regulated cornified cell envelope precursor proteins [29]
1.91	0.0311	SPRR2G	Small proline‐rich protein 2G	
−1.8	0.0486	CRNN	Cornulin	
3.07	0.0424	KPRP	Keratinocyte proline‐rich protein	Keratinocyte proline‐rich protein deficiency in atopic dermatitis leads to barrier disruption [30]
Matrix metallopeptidases				
−2.38	0.0163	MMP3	Matrix metallopeptidase 3	Matrix metalloproteinase‐3 is the key effector of TNF‐α‐induced collagen degradation in skin [31]
−2.82	0.0437	MMP1	Matrix metallopeptidase 1	Matrix metalloproteinase‐1 is the major collagenolytic enzyme responsible for collagen damage in UV‐irradiated human skin [32]
−1.78	0.021	MMP14	Matrix metallopeptidase 14 (membrane‐inserted)	
Retinoid metabolism				
1.71	0.0241	CRABP1	Cellular retinoic acid‐binding protein 1	
1.55	0.0316	RDH13	Retinol dehydrogenase 13 (all‐trans/9‐cis)	Catalyses conversion of retinol to retinal
−1.59	0.0466	DHRS3; MIR6730	Dehydrogenase/reductase (SDR family) member 3; microRNA 6730	
−1.65	0.0248	ALDH1A3	Aldehyde dehydrogenase 1 family, member A3	
−1.71	0.0328	RARB	Retinoic acid receptor, beta	
−1.73	0.017	PALM	Paralemmin	
−1.76	0.0037	APOE	Apolipoprotein E	
−2.08	0.0034	RDH11	Retinoldehydrogenase 11 (all‐trans/9‐cis/11‐cis)	
−1.76	0.0231	AKR1C1	Aldo–keto reductase family 1, member C1	Reduces retinaldehyde to retinol conversion, limiting availability of retinoic acid

### Human explants (Collagen IV)

**FIGURE 5:**
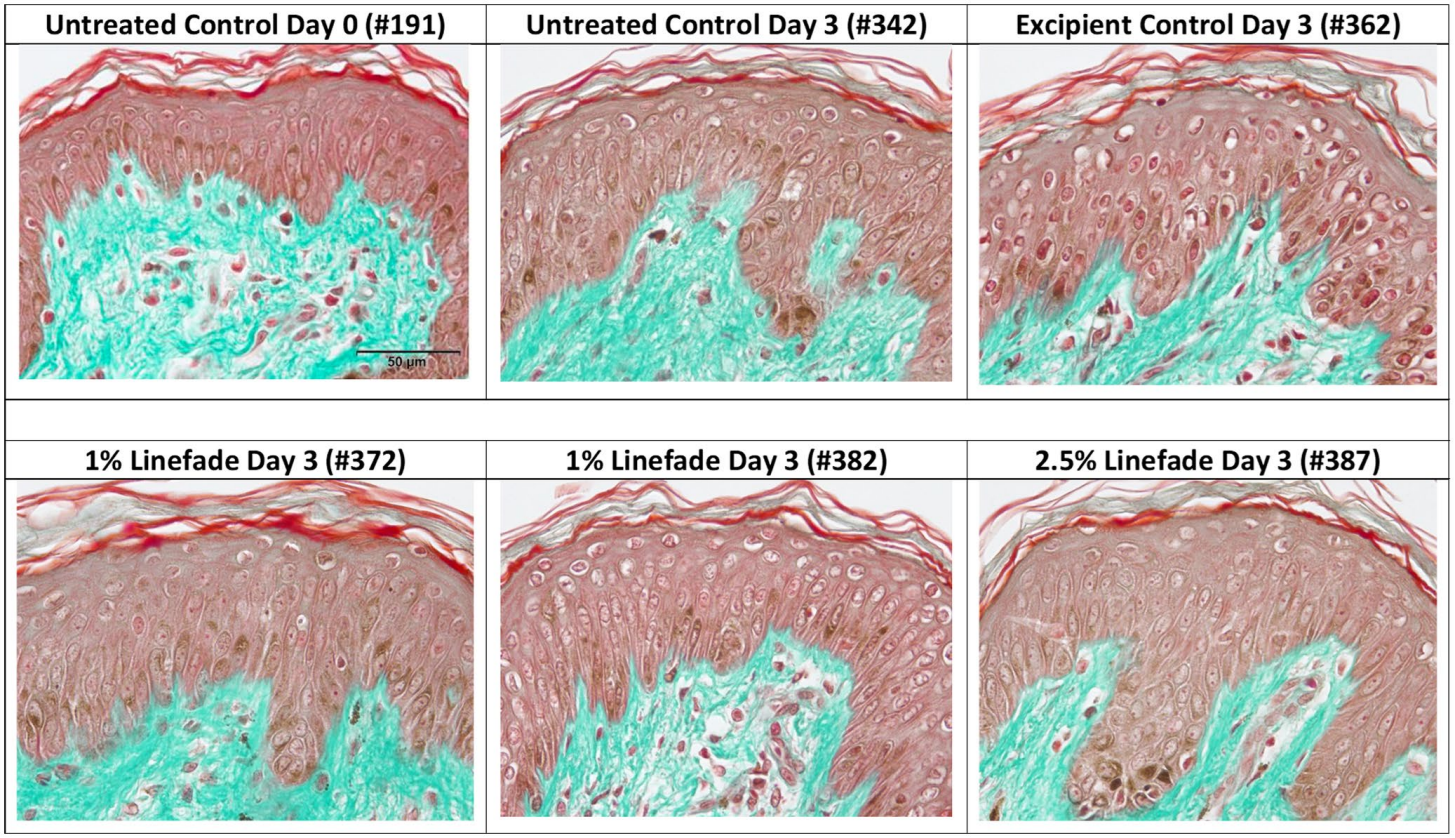
Tissue morphology of day 0 and day 3 batches

An ex vivo study was performed using human skin explants to further investigate whether Linefade could increase collagen IV synthesis in the ex vivo model. On days 0 and 3, tissue morphology was observed to be good in the epidermis and papillary dermis in the untreated control explants. On day 3, the excipient‐treated control (CCT) samples showed slight alteration in the epidermis. The samples treated with 1% Linefade showed slight alteration in the epidermis while treatment with 2.5% Linefade did not result in any modification when compared with the untreated control group. On day 3, the samples treated with 1% Linefade did not show any modification in the epidermis and papillary dermis, while treatment with 2.5% Linefade led to a slight improvement in tissue viability in the epidermis when compared to the excipient control group as shown in Table 4 and Figure 5.

**TABLE 4:** Tissue morphology microscopic assessments of the epidermal and dermal structures of all explant batches is shown here

Explant batch	Tissue morphology observations	
	Epidermis	Dermis
Untreated Control Day 0	G	G
Untreated Control Day 3	FG	G
Excipient Control Day 3	SA	G
1% Linefade Day 3	SA	G
2.5% Linefade Day 3	FG	G

Note

Morphology legend: G = good, FG = fairly good, VSA = very slightly altered, SA = slightly altered, MA = moderately altered, FCA = fairly clearly altered, CA = clearly altered, VCA = very clearly altered.

Immunostaining revealed a significant increase in collagen IV along the DEJ as compared to untreated control and excipient‐treated control groups on day 3.

**FIGURE 6:**
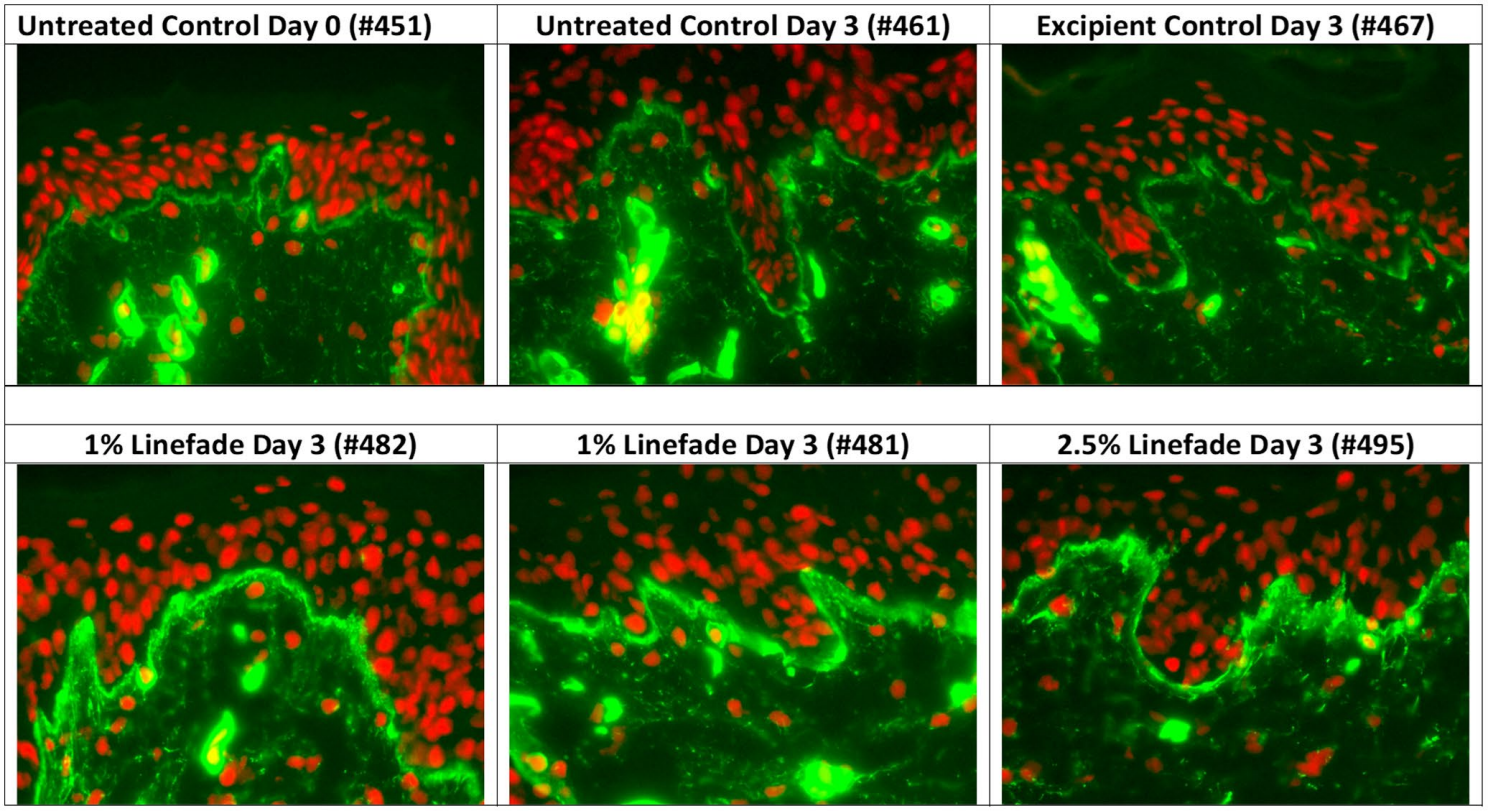
Effects of Linefade on collagen IV synthesis in skin explants vs. untreated and excipient‐treated controls. Collagen IV was detected by immunostaining using specific antibodies. Number of explants per condition = 3

**FIGURE 7:**
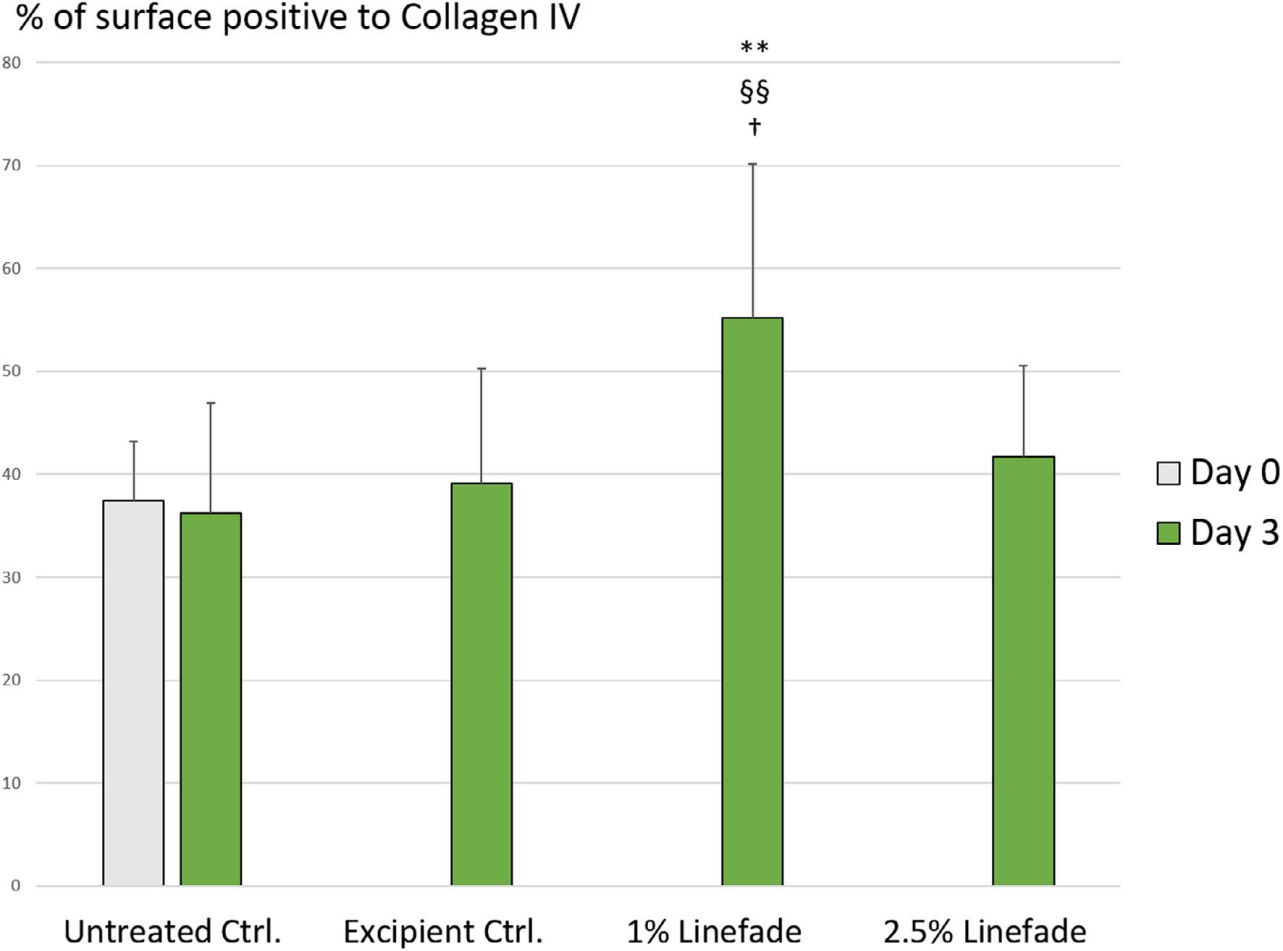
Quantification of collagen expression levels in Figure 6 (*n* = 9 with 3 images per explant) Data are presented as mean ± standard deviation. Calculated proportion of the surface to collagen IV along the DEJ. Treated samples vs. untreated control (Day 0); ** for *p*‐value < 0.01. Treated samples vs. untreated control (Day 3); §§ for *p*‐value < 0.01. Treated vs. excipient control (Day3); † for *p*‐value < 0.05

Image analysis of collagen IV was analysed in terms of percentage of collagen IV surface. It was observed that on day 3, 1% Linefade showed a significant increase in collagen IV along the DEJ (52%; *p*‐value <0.01) as compared to untreated control group. Furthermore, treatment with 1% Linefade resulted in a significant increase in collagen along the DEJ (41%; *p*‐value <0.05) as compared to excipient‐treated control group. Treatment with 2.5% Linefade did not produce a statistically significant result (Figures 6 and 7).

## DISCUSSION

Skin ageing is a continuous process, accelerated by oxidative stress and governed by intrinsic age‐related metabolic changes. PPAR‐α is a key regulator of this process via prevention of damage and control of repair after exposure to endogenous or environmental stressors [33]. PPAR‐α mediates the transcription of lipid metabolizing genes and regulates the genes involved in maintaining the redox homeostasis, energy metabolism and integrity of tissues [34].

Altered DEJ is a characteristic of skin ageing. Type IV collagen, a fundamental constituent of the DEJ, provides a scaffold for other macromolecules and plays a key part in maintaining mechanical steadiness and resilience [35]. Therefore, the identification of compounds that can increase the level of collagen IV in DEJ is advantageous for managing changes associated with skin ageing.

To elucidate the mechanism of action of Linefade on skin ageing, in silico, in vitro and ex vivo studies were performed. In silico docking analyses showed that Linefade forms H‐bonds with CYS‐276, SER‐280, CYS‐275, THR‐279, MET‐330, MET‐355 and GLN‐277 in the PPAR‐α binding pocket. Utsugi et al. (2019) proposed a synthetic covalent PPAR agonist based on the unique character of the PPAR ligand‐binding pocket (LBP) that can be possessed by different ligands owing to its expansive depth coupled with the synergistic activation of the transcriptional switch of PPAR [36]. Takeda et al. (2018) showed that tiliroside, a PPAR‐α agonist, can stimulate ceramide‐related enzyme expression and increase PPAR‐α expression. Interestingly, in this study, 10% tiliroside delivered as part of a complex mixture obtained from strawberry extractions resulted in superior bio‐activity as compared to equivalent concentrations of the purified tiliroside [37]. PPAR‐α signalling plays a crucial role in skin development and barrier formation through induction of keratinocyte proliferation and differentiation [38, 39]. In the present study, we found that Linefade activates PPAR‐α with high potency, as compared to the positive control GW590735, in in vitro luciferase reporter gene assay. This finding may be attributed to the Y‐shaped binding cavity of PPAR containing several sub‐pockets that enable co‐binding for a mixture of compounds, such as Linefade.

Since hydroxystearic acids have previously been shown to act as both PPAR agonists and stimulators of collagen synthesis, in this study, we utilized a glyceryl monoricinoleate (glycerol 12‐hydroxyoleate) prepared from the esterification of plant‐derived ricinoleic acid (a hydroxy acid) and glycerine. This ester was used to solubilize the oleanolic acid and other constituents of an olive leaf extraction, thereby creating a liquid mixture of PPAR agonists. Our modelling work demonstrated a potential for cooperative binding between the two major constituents (OA + GMR) of Linefade. Taken together, these compounds exhibited seven common interactions with the 11 amino acid residues interacting with the synthetic control PPAR‐α agonist GW590735.

To further elucidate the mechanism of action of Linefade, transcriptome analysis was performed in the full‐thickness reconstituted human skin (EpiDermFT) model. Consistent with PPAR activation, we found significant modulation of cornified envelop precursor genes, including LCE (late cornified envelop) genes, S100 family genes, SPRR (small proline‐rich proteins) and other regulators of epidermal differentiation (such as cornulin and keratinocyte proline‐rich protein). Furthermore, we observed downregulation of collagen degrading matrix metalloproteinase‐1 and matrix metalloproteinase‐3. PPAR activation is known to suppress TNF‐α and these inflammatory markers.

Upon screening via Wikipathways, we found significant modulation of the Nrf2 and PPAR pathways. Both transcription factors play key roles in establishing a cellular antioxidative defence system and are known to crosstalk [40, 41]. Lastly, we found modulation in 10 genes involved in retinoid metabolism. While the downregulation of AKR1C1 and upregulation of cellular retinoic acid‐binding protein 1 and retinol dehydrogenase 13 may support increased availability of retinoic acid at the retinoid receptors, the downregulation of retinaldehyde dehydrogenase 3, dehydrogenase/reductase 3 and retinol dehydrogenase 11 would reduce RA availability. This retinoid pathway modulation may be attributed to the inhibitory action of oleanolic acid on AKR1B10.

Altered DEJ in aged skin shows diminished connectivity in surface area [42]. Loss of DEJ surface range contributes to the expanded skin delicacy related to age and leads to diminished connectivity between the dermal and the epidermal layers [43]. We assessed the type IV collagen levels in human dermal fibroblasts and human skin explants. Our results indicate that Linefade treatment significantly increased the levels of collagen type IV in in vitro and ex vivo models. We also observed slight alterations in cellular morphology after 3 days of treatment with the excipient CCT ex vivo. It is noteworthy that treatment with 2.5% Linefade did not lead to any undesirable alterations in the tissue morphology in excipient‐treated samples; however, this concentration also did not induce a statically significant collagen IV expression as was observed with the 1% treatment. This finding indicates that Linefade may exhibit a protective activity, which counters the alterations induced by CCT treatment, yet the 2.5% dose may interfere with collagen deposition in the explants model. Glyceryl monoricinoleate and dimethyl isosorbide have surfactant and solvency properties which may explain why this 2.5% group showed several explants with increased collagen IV while others explants in this group did not show increases in collagen IV staining.

Even though PPAR‐α agonists can stimulate the expressions of serine palmitoyltransferase (SPT) 2 and ceramide synthase (CerS) 3, and improve the skin barrier function and moisture retention in ceramide‐deficient skin condition, Linefade was not observed to modulate the expression of serine palmitoyltransferase (SPT) 2 and ceramide synthase (CerS) 3. This may suggest that PPAR ligand activation may occur with partial, full or varied configurations altering its ability to mediate gene transcription. Furthermore, it is unclear whether the agonist‐bound PPAR‐α can directly transactivate collagen IV gene without promoter analysis. PPAR‐α might indirectly increase collagen IV synthesis by modulating other transcription factors. Lastly, we have to consider that about 0.4% of the Linefade complex may contain a variety of other alcohol‐soluble compounds including maslinic acids, oleuropein, oleuropeoside, ligustroside and hydroxytyrosol. While, at the experimental dilutions, these compounds are found at negligible concentrations, we cannot ignore a possibility that these trace compounds may contribute to bio‐activities especially given that Takeda et al. (2018) had observed a strawberry extract standardized to 10% tiliroside (PPAR agonist) outperform a highly purified form of tiliroside.

## CONCLUSION

Linefade contains 2% olive leaf extract solids dissolved in glyceryl monoricinoleate and dimethyl isosorbide. Linefade binds to PPAR‐α ligand‐binding domain and induces transcriptional activation, wherein it modulates 280 genes and induces collagen IV synthesis in vitro and ex vivo. Future studies must focus on the potential applications of Linefade, such as formulation of skin care products to manage the visible and mechanical changes of ageing skin, adjuvant‐type applications for OTC skin protectants and therapeutic applications in cases of skin blistering and separation of the epidermis from the dermis. The key insight of our study was that Linefade, a lipidic mixture, acts as a PPAR‐α agonist and activates collagen IV biosynthesis in the skin, thereby increasing the pool for replacement and inhibiting changes related to skin ageing. Further studies will determine whether Linefade effects are translated into clinical benefits in aged and diseased skin.
